# Unspecific Peroxygenases for the Enzymatic Removal of Alkyl Protecting Groups in Organic Synthesis

**DOI:** 10.1021/acscatal.5c06385

**Published:** 2025-09-26

**Authors:** Lina A. Csechala, Maximilian Wutscher, Verena Scheibelreiter, Stefan Giparakis, Ina Menyes, Thomas Bayer, Christian Stanetty, Florian Rudroff, Uwe T. Bornscheuer

**Affiliations:** † Department of Biotechnology & Enzyme Catalysis, Institute of Biochemistry, 26552 University of Greifswald, Felix-Hausdorff-Str. 4, Greifswald 17489, Germany; ‡ Institute of Applied Synthetic Chemistry, 27259 TU Wien, Getreidemarkt 9, Vienna 1060, Austria

**Keywords:** biocatalysis, ether cleavage, *O*-dealkylation, protection group chemistry, unspecific peroxygenases

## Abstract

Selective protection and deprotection of hydroxyl groups is pivotal in multistep organic synthesis to circumvent undesired side reactions. Alkyl ethers are highly stable and atom-economic protecting groups (PGs), but demand harsh and hazardous conditions for removal, limiting their utility. Consequently, there is a high demand for biocatalysts as milder, selective, and scalable alternatives, which can be met by a class of heme-thiolate enzymes: unspecific peroxygenases (UPOs). Herein, we report the identification of UPO23 in a commercial enzyme panel as a robust biocatalyst for *O*-dealkylation reactions. UPO23 exhibited a broad substrate scope and efficiently removed methyl, ethyl, propyl, or allyl groups from protected primary, secondary, tertiary, and benzylic alcohols under ambient conditions. Mechanistic investigations revealed dual reaction pathways for UPO23, hydroxylating either the α-carbon of the alkyl chain of the PG or the substrate scaffold, explaining the formation of deprotected target alcohols as well as further oxidized products. Optimized reaction conditions reduced reaction times from 4 h to 15 min for methyl protected key substrates. Preparative scale reactions with protected benzyl ethers yielded up to 92% of the isolated alcohol products. These findings highlight the versatility of UPO23 and offer scalable, environmentally benign, and enzyme-based deprotection strategies for multistep organic synthesis.

## Graphical abstract

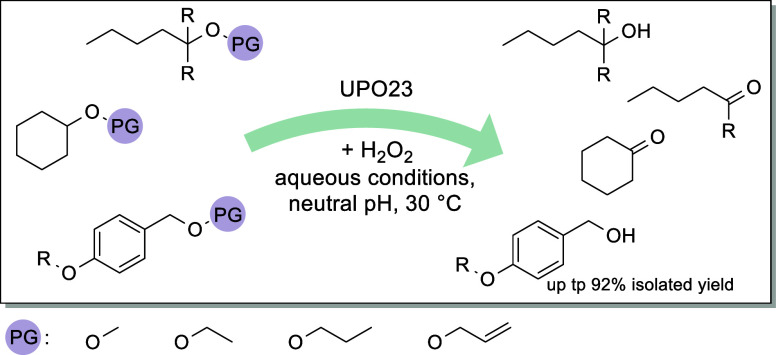

## Introduction

The protection and deprotection of functional groups is essential during multistep organic synthesis to prevent undesired side-reactions. The ability to particularly protect and deprotect alcohol groups has become increasingly significant due to their prevalence in natural products and the variety of transformations that involve hydroxyl-containing intermediates.^1^ The choice of protecting groups (PGs) as well as strategies for their specific removal are decisive factors in the successful realization of complex synthetic schemes.^2–4^ Therefore, PGs have to be carefully chosen based on their size, robustness, and potential modification of other functional groups. Common PGs for alcohols include esters and different ethers, such as various benzyl or silyl ethers.^2,5,6^ Small alkyl groups (e.g., methyl, ethyl, propyl, and allyl groups) are viable alternative PGs for alcohols. They are small in size, cheap, more atom efficient, and easy to introduce.^2,7^ Once installed, the resulting ether bonds are highly stable across a wide range of reaction conditions without the risk of premature deprotection.^8^ Moreover, their small size minimizes steric hindrance, which is advantageous compared to the larger, commonly used PGs (e.g., *t*-butyldimethylsilyl ether (TBDMS)). However, the stability of alkyl ether bonds becomes a drawback during deprotection, which frequently requires elevated temperatures and reactive reagents such as strong (Lewis) acids (e.g., AlCl_3_ or BBr_3_) or (heavy) metal catalysts.^9–12^ These conditions may affect labile molecular scaffolds and other sensitive functional groups, limiting the use of small alkyl PGs to mask hydroxyl groups.^13,14^ Moreover, the cleavage of aliphatic alkyl ethers is problematic due to potential side reactions like the elimination of the activated alkoxy group. To address the shortcomings of chemical deprotection methods, the enzyme-catalyzed removal of PGs, and more specifically alkoxy groups, is an appealing alternative due to the inherent properties of biocatalysts, which operate under mild reaction conditions, including neutral pH and ambient temperatures, in aqueous buffer systems.^15–17^ Importantly, enzymes exhibit high chemo-, regio-, and stereoselectivity, allowing the specific cleavage of bonds at reduced off–target activities. This is preferable if multiple, chemically similar PGs are present in the same molecule.^18^ Together, the use of enzymes for deprotection offers safer and environmentally benign procedures by circumventing the need for harsh reaction conditions and hazardous reagents.^15,19^

Two enzyme classes that can perform the cleavage of alkyl ether bonds are cytochrome P450 monooxygenases (CYPs) and unspecific peroxygenases (UPOs).^20–22^ Both are heme-thiolate proteins that have been shown to catalyze a variety of oxyfunctionalization reactions, including hydroxylations, epoxidations, sulfoxidations, *N*-oxidations, and, importantly, *O*- and *N*-dealkylations.^23–25^ During *O*-dealkylations, these enzymes hydroxylate one of the carbons adjacent to the oxygen in an ether bond, forming an unstable hemiacetal intermediate that spontaneously decomposes to yield an alcohol and an aldehyde or ketone. The specific products depend on which side of the ether bond was initially hydroxylated.^26^ CYPs have a broad substrate scope and depend on molecular oxygen (O_2_) as electron donor, as well as reduced nicotinamide adenine dinucleotide (phosphate) (NAD­(P)­H) as cofactor. Additionally, certain classes of CYPs require redox partner proteins for the electron transfer. This is one limitation affecting their usability and scalability for industrial applications.^27,28^ In contrast, UPOs operate independently of additional cofactors and utilize hydrogen peroxide (H_2_O_2_) or organic hydroperoxides as oxygen donor and electron acceptor. First discovered in 2004,^29^ UPOs have demonstrated a broad substrate scope and were used for aliphatic and aromatic ring oxidations in the context of the biobased degradation of xenobiotics,^30–35^ or the production of fine chemicals such as grevillic acid.^36^ This makes the use of UPOs more practical and cost-effective in comparison to CYPs and enabled their implementation in industrial large-scale processes recently.^18,31,33,37^

Due to the superior features of UPOs and the high demand in biocatalytic deprotection strategies for ether-protected hydroxyl groups, we investigated a commercial panel of UPOs toward its *O*-dealkylation activities. To date, only a few UPOs have been reported to cleave ether bonds, such as *Aae*UPO from *Agrocybe aegerita* ^38,39^ and variants,^40^ besides others.^41,42^ These examples from the literature predominantly describe the potential of UPOs to cleave ether bonds but are mostly limited to screening, and simple GC–MS data. A systematic investigation for their synthetic application as a biocatalytic deprotection reagent is so far missing. In this work, we identified an UPO, selectively acting on structurally different molecular scaffolds containing methyl-, ethyl-, and even larger alkyl ethers. These findings greatly expand the enzymatic toolbox for mild and scalable deprotection strategies in organic synthesis as discussed in the following.

## Results and Discussion

### Substrate Library Creation and Screening of UPOs

To test the deprotection potential across a broad spectrum of substrate classes and PGs, a substrate library was created, containing aliphatic primary, secondary, and tertiary alcohols, as well as cyclic, phenolic, and benzylic alcohols, each protected as methyl (**a**), ethyl (**b**), propyl (**c**), or allyl ethers (**d**) (Scheme 1).

**Scheme 1:**
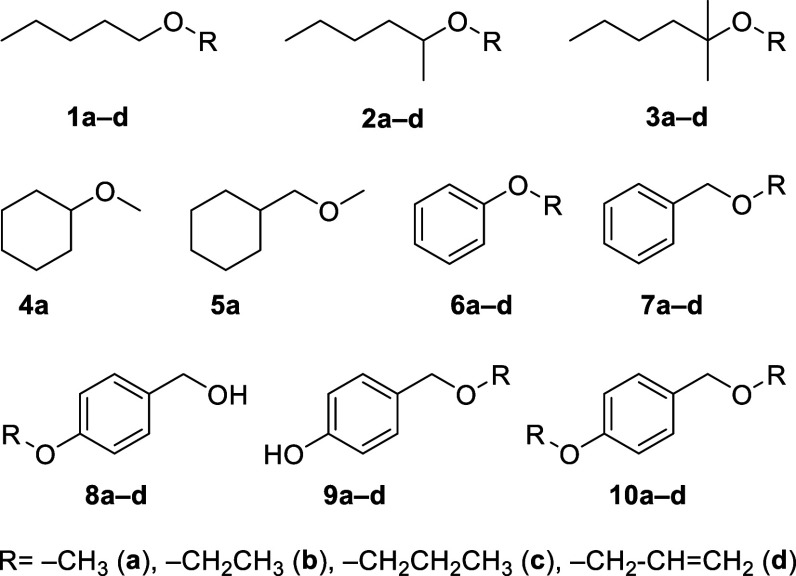
Substrate Library of *O*-Protected Alcohols Investigated in This Study; Molecular Scaffolds (**1–10**) Included Aliphatic, Alicyclic, and Aromatic Compounds, Containing Primary, Secondary, and Tertiary Alcohol Groups that are Protected as Methyl (**a**), Ethyl (**b**), Propyl (**c**), or Allyl (**d**) Ethers

To identify promising enzyme candidates, a panel of 44 UPOs (Aminoverse B.V., Nuth, The Netherlands) was initially screened against nine methyl-protected alcohol substrates (**1–9a**). The *O*-demethylation activity was assessed by the Purpald assay, a colorimetric assay in which the Purpald reagent reacts with the formaldehyde produced during the UPO-catalyzed reaction, forming a purple dye (see Figure S1).^43^ UPOs yielding the highest absorbance (within the top 25%) in the Purpald assay were selected as promising candidates. In many UPO-catalyzed reactions, unidentified products were formed that did not correspond to the desired alcohol or overoxidized products (e.g., aldehydes/ketones and carboxylic acids).^25^ These side-products were not further identified, since our focus was on the discovery of robust deprotection candidates. Among the enzymes that showed *O*-demethylation activity on the investigated substrates, UPO23 exhibited the broadest substrate scope (Figure S1), yielding the expected demethylated products as confirmed by calibrated GC-FID (Figure 1). Hence, UPO23 (very recently described as *Atu*UPO where it showed hydroxylation activity on 4-propylguiacol)^44^ was selected and its *O*-dealkylation activity studied against the extended substrate library, aiming at the removal of methyl, ethyl, propyl, or allyl PGs. Satisfyingly, UPO23 also cleaved different ether bonds in structurally different target substrates (Figure 1).

**Figure 1:**
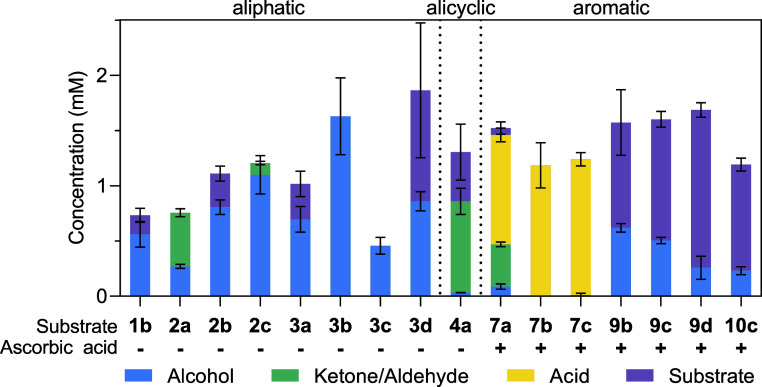
Initial screening for the *O*-dealkylation activity of UPO23. Reactions were performed at 30 °C with shaking at 650 rpm for 4 h in 100 mM tricine buffer (pH 7.5) with 2 mM substrate, 0.1 mg mL^–1^ lyophilized UPO23, with (+) or without (−) ascorbic acid. H_2_O_2_ was added in 30 min intervals (up to 8 mM). Samples were analyzed by calibrated GC-FID or HPLC-UV/vis as described below. Concentration of reaction products are shown as mean values ± standard deviation (SD) from reaction replicates (*n* = 3).

While 1-ethoxypentane (**1b**) was exclusively dealkylated to 1-pentanol (28% GC yield), the reaction with 2-methoxyhexane (**2a**) yielded the ketone 2-hexanone as the main product (24% GC yield) after 4 h reaction time. The formation of 2-hexanone was unexpected and could either indicate subsequent overoxidation or be caused by the activation of the ether bonds’ alternate α-carbon. This was further evaluated as described below. Substrates with longer PGs (**2b** and **2c**) were converted to 2-hexanol (43 and 55% GC yield, respectively). The cleavage of tertiary ethers is particularly noteworthy, considering the challenges of chemical deprotection methods for tertiary and other sterically hindered alcohols.^13^ While all studied PGs were cleaved from different target substrates, 2-ethoxy-2-methylhexane (**3b**), protected with an ethyl group, yielded the highest amount of the deprotected alcohol (82% according to calibrated GC-FID). UPO23 also showed moderate to good activity on **3a**, **3c**, and **3d**, containing methoxy, propoxy, and allyloxy groups (35, 23, and 48% GC yield of tertiary alcohol, respectively). The alicyclic methoxycyclohexane (**4a**) was also converted by UPO23, resulting in the formation of cyclohexanone with a yield of 41% according to calibrated GC-FID. Only trace amounts of cyclohexanol were detected, indicating that the reaction could be selective toward the formation of the ketone as discussed below. Altogether, UPO23 readily catalyzed the deprotection of primary (**1b**), secondary (**2a–c**, **4a**), and tertiary alcohols (**3a–d**) from aliphatic substrates, except for (methoxymethyl)­cyclohexane (**5a**), which was not converted and, therefore, not further investigated.

Motivated by these findings, the potential of UPO23 to deprotect aromatic ether substrates was investigated next. Ascorbic acid was added to all reactions as a radical scavenger to suppress peroxidase activity by quenching transiently formed phenoxy radicals.^45,46^ While UPO23 did not catalyze the deprotection of alkyl aryl ethers with different PGs (**6a–d**, **8a–d**), consistent with recent reports about *Atu*UPO,^44^ the enzyme did cleave benzyl ethers (**7**, **9**, and **10**). For substrates with a benzyl ether moiety only, the reaction predominantly yielded the corresponding carboxylic acid (Figure 1). While benzyl methyl ether (**7a**) was converted to a mixture of benzaldehyde and benzoic acid (19 and 50% HPLC yield, respectively), benzyl ethyl ether (**7b**) and benzyl propyl ether (**7c**) yielded benzoic acid as the main product (59 and 61%, respectively, according to calibrated HPLC-UV/vis). In contrast, only the target alcohol was observed for benzyl ethers substituted with a hydroxyl group in *para* position (**9a–d**), implying that a substituent on the aromatic ring suppresses the formation of aldehydes and carboxylic acids. The highest yield was observed with 4-(ethoxymethyl)­phenol (**9b**), which was exclusively converted to the corresponding alcohol with an average GC yield of 31%. Substrates with longer PGs (**9c** and **9d**) were also dealkylated to 4-(hydroxymethyl)­phenol (25 and 13% GC yield, respectively). Finally, substrates containing both, a protected phenol and a protected benzyl alcohol in *para* position (**10a–d**), were studied. Consistent with the previous results, in the reaction with 1-ethoxy-4-(ethoxymethyl)­benzene (**10c**), the benzyl ether was selectively cleaved to form the target alcohol, while the phenol group remained protected, yielding 12% of (4-propoxyphenyl)­methanol according to calibrated GC-FID. These data demonstrate a limitation of UPO23 for benzyl ethers with an additional substituent since they were converted at lower yields compared to substrates **7b–c**.

Together, these results confirm the versatility of UPO23, exhibiting *O*-dealkylation activity toward our extended library of ether-protected alcohol substrates.

### Evaluation of Overoxidation and Potential Reaction Pathways

After observing the formation of significant amounts of aldehydes, ketones, and carboxylic acids in reactions with substrates **2a**, **4a**, **7a**, **7b**, and **7c** in the presence of an excess of H_2_O_2_ and after long incubation times, we aimed at understanding potential reaction pathways (Scheme 2) in order to optimize reaction conditions toward the formation of target *O*-dealkylated alcohol products. Therefore, we subjected 2 mM of these substrates, added limiting amounts of H_2_O_2_ (1 mM), and performed UPO23-catalyzed reactions for up to 10 min (Figure 2A).

**Scheme 2:**
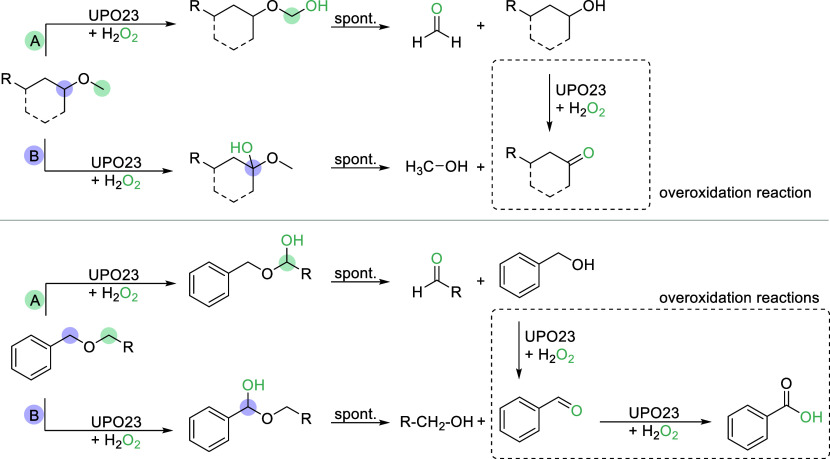
Putative UPO Reaction Pathways Promoting Overoxidation; UPOs Activate the α-Carbon of the Short Alkyl-Group of the PG (Pathway A) or the α-Carbon of the Substrate Scaffold, Resulting in Different *O*-Dealkylation Products; Further Overoxidations of Alcohols and Aldehydes are Possible; Different Reaction Routes are Shown for the Deprotection of Aliphatic, Secondary Alcohols (Top; e.g., **2a** and **4a**) and Ether-Protected Benzyl Alcohols (Bottom)

**Figure 2:**
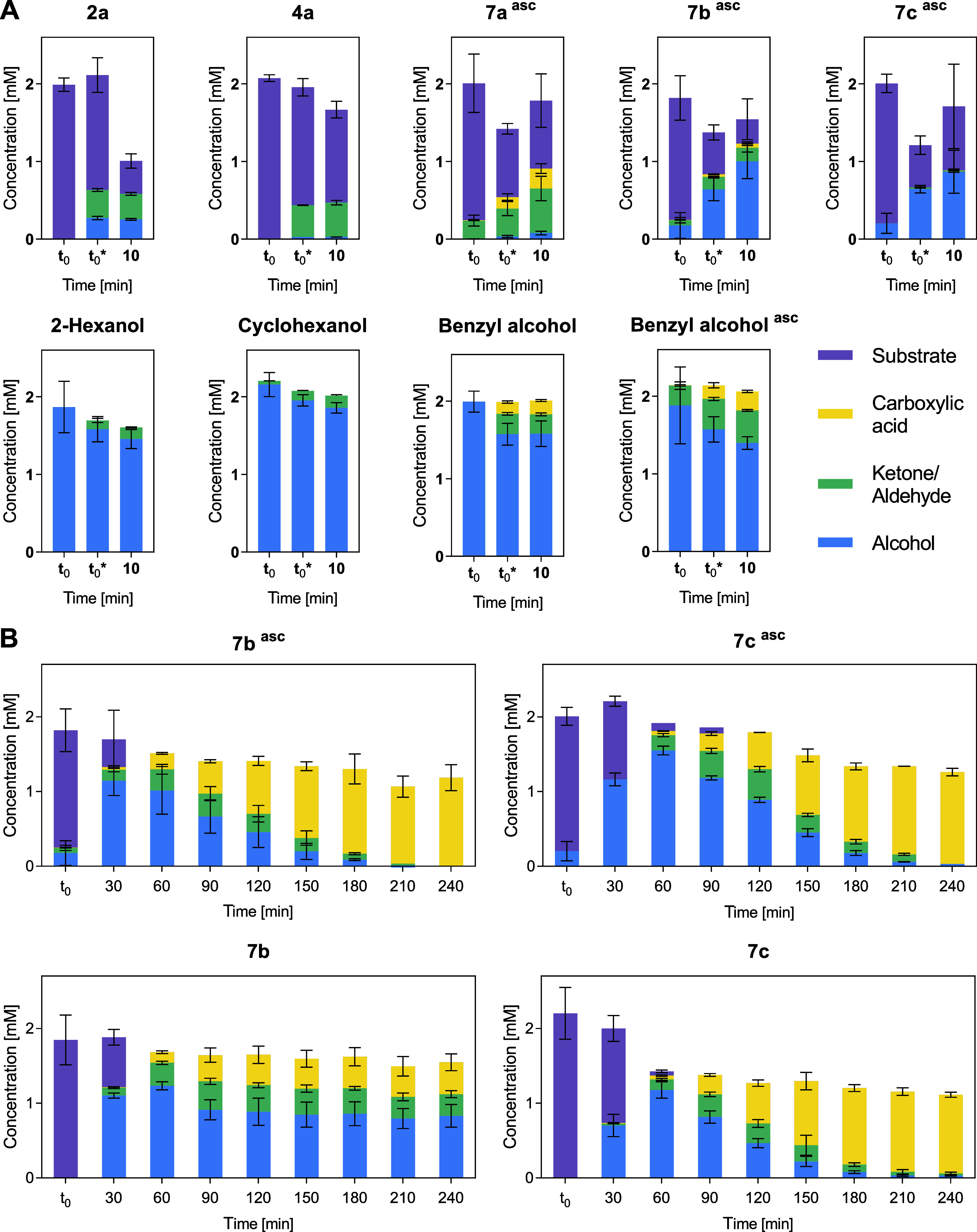
Reaction progress of *O*-dealkylation reactions catalyzed by UPO23. Reactions were performed at 30 °C with shaking in 100 mM tricine buffer (pH 7.5), containing 2 mM substrate, 2 mM ascorbic acid as indicated (^asc^), and 0.1 mg mL^–1^ lyophilized UPO23. H_2_O_2_ was added up to (A) 1 mM final concentration (10 min reaction time) or (B) 8 mM (added in 30 min intervals up to 4 h reaction time). Samples were analyzed by calibrated GC-FID or HPLC-UV/vis as described below. Concentration of reaction products are shown as mean values ± SD from reactions replicates (*n* ≥ 3); *t*_0_ and *t*_0_* indicate composition of reaction mixtures before and after the addition of H_2_O_2_, respectively.

For substrates **7b** and **7c**, benzyl alcohol was the main product, detected immediately after the addition of H_2_O_2_ (*t*_0_*), with only traces of benzaldehyde and benzoic acid observed for **7b**. Monitoring the reaction progress revealed that **7b** and **7c** were converted to the target benzyl alcohol with 51 and 77% HPLC yield, respectively, after 1 h (Figure 2B). With an excess of H_2_O_2_ and a prolonged reaction time, UPO23 catalyzed the overoxidation of benzyl alcohol, leading to the formation of benzaldehyde and benzoic acid. After 3–4 h, benzoic acid was the major product (Figure 2B). These results support a sequential oxidation pathway, in which benzyl alcohol is initially formed via ether cleavage, oxidized to the aldehyde and further to the corresponding carboxylic acid (Scheme 2, bottom, pathway A). Notably, the addition of ascorbic acid did not prevent the formation of overoxidation products and appeared to enhance it. While ascorbic acid is typically used as a radical scavenger to suppress peroxidase activities, which can promote (over)­oxidation reactions, our results suggest a more complex role.^36,47^ Deng et al. proposed that small-molecule reductants such as ascorbic acid enable a catalytic pathway, allowing UPOs to utilize O_2_ instead of H_2_O_2_.^48^ Similarly, a recent study demonstrated enhanced activity of several UPOs (including *Atu*UPO) in the presence of ascorbic acid.^44^ Evidence for this O_2_/reductant-dependent catalytic route with UPO23 can be found in the conversion of benzyl alcohol. The formation of benzaldehyde was observed at *t*_0_, even before the addition of H_2_O_2_, but only in the presence of ascorbic acid (Figure 2A). These results indicate that the function of ascorbic acid as a reductant outcompetes the function as a radical scavenger, leading to the observed accelerated overoxidation.^44^

In the absence of ascorbic acid (Figure 2B), the overoxidation was only catalyzed to a small extent in reactions with substrate **7b**. After 90 min, only 19% benzaldehyde and 18% benzoic acid were detected, according to calibrated HPLC-UV/vis, and the yields did not change significantly as the reaction progressed. After 4 h, benzyl alcohol was the main product (42% HPLC yield), which is in contrast to the exclusive formation of benzoic acid observed in the presence of ascorbic acid (59% HPLC yield). In reactions with substrate **7c**, benzoic acid remained the main product after 4 h under both conditions. However, the observed HPLC yield decreased from 61 to 53% in the absence of ascorbic acid (Figure 2B).

In comparison, reactions with the aliphatic **2a**, the alicyclic **4a**, and the benzylic **7a** showed the immediate formation of the corresponding ketone, aldehyde, or carboxylic acid upon the addition of H_2_O_2_ (Figure 2A). However, in a control experiment, the conversion of 2-hexanol, cyclohexanol, and benzyl alcohol by UPO23 within 10 min reaction time was slow (Figure 2A). The lack of transient alcohol products, together with the slow overoxidation, potentially suggests an alternative reaction mechanism we aimed to investigate next.

Commonly, UPOs activate the α-carbon of the short alkyl-group of the PG, resulting in the formation of a deprotected alcohol and a short-chain aldehyde.^26,38^ Further overoxidation reactions can follow, in which the alcohol is oxidized to a ketone or aldehyde; the latter can be further converted to the corresponding carboxylic acid (Scheme 2, pathway A).^49,50^

Alternatively, the α-carbon of the substrate scaffold can be activated, yielding a deprotected aldehyde or ketone and a short-chain alcohol.^38^ Overoxidations can also occur here (Scheme 2, pathway B). To determine the favored reaction pathway for selected substrates, it was analyzed whether short aldehydes were released during transformations catalyzed by UPO23. Reactions were performed under limiting H_2_O_2_ conditions (0.25 mM), quenched after 10 min, and derivatized with 2,4-dinitrophenylhydrazine (2,4-DNPH).^51^

Since experimental results and products formed from substrates **7b** and **7c** suggest reaction pathway A (Scheme 2, bottom), we validated the 2,4-DNPH derivatization method with these substrates. As expected, *O*-dealkylation reactions released acetaldehyde and propionaldehyde, respectively (Figure 3). In reactions with **7b**, low amounts of benzaldehyde were detected, which is consistent with previous results and could indicate a minor contribution of pathway B (Scheme 2, bottom).

**Figure 3:**
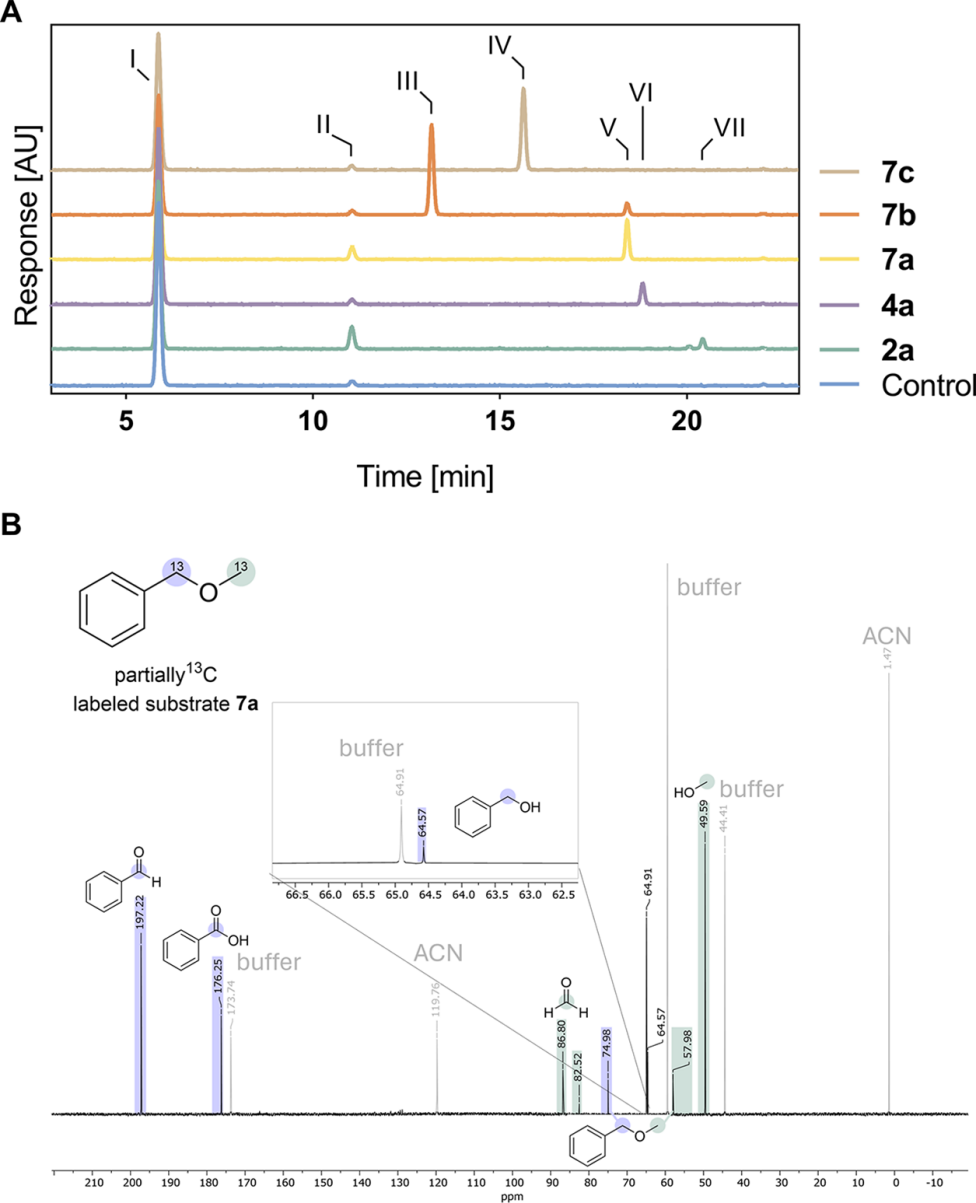
Evaluation of reaction pathways. (A) uHPLC-analysis of released aldehydes, derivatized with 2,4-DNPH. Reactions contained 2 mM substrate and 0.1 mg mL^–1^ lyophilized UPO23 in 100 mM tricine buffer (pH 7.5) and were initiated with limiting H_2_O_2_ (0.25 mM). After 10 min incubation at 30 °C with shaking, the reactions were quenched and derivatized with 2,4-DNPH. Reactions were performed in triplicates. Exemplary stacked chromatograms are displayed and show a negative control without substrate (blue), **2a** (green), **4a** (purple), **7a** (yellow), **7b** (orange), and **7c** (ochre). Full chromatograms are displayed in Figure S2. 2,4-DNPH derivatization products were labeled as follows: **I**: 2,4-DNPH only; **II**: formaldehyde; **III**: acetaldehyde; **IV**: propionaldehyde; **V**: benzaldehyde; **VI**: cyclohexanone; **VII**: 2-hexanone. (B) NMR of ^13^C experiment. The reaction was performed at 30 °C with stirring in 100 mM tricine buffer (pH 7.5), containing 2 mM ^13^C-labeled substrate **7a** and 0.1 mg mL^–1^ lyophilized UPO23. H_2_O_2_ was added up to 2 mM final concentration (1 mM added at 0 and 10 min, total reaction time 20 min).

No formaldehyde was detected in reactions with substrate **4a** (Figure 3), which proves that the hydroxylation occurred exclusively on the cyclohexyl moiety, as shown for pathway B (Scheme 2, top). Reactions with substrates **2a** and **7a** only yielded low amounts of formaldehyde (Figure 3), suggesting that pathway A is partially involved in the catalyzed reaction (Scheme 2, top). Nonetheless, the low levels of formaldehyde indicate that the main reaction progresses via pathway B, involving the hydroxylation of the α-carbon of the main chain. To further validate the proposed pathway B, ^13^C-labeled **7a** was synthesized in this work and investigated in reactions with stoichiometric H_2_O_2_. The reaction products were analyzed by nuclear magnetic resonance (NMR) spectroscopy. The ^13^C NMR results showed the formation of significant amounts of ^13^C-labeled methanol and only traces of ^13^C-labeled formaldehyde (Figure 3B). These results confirm that the reaction is mainly proceeding via pathway B (Scheme 2, bottom).

Interestingly, an extension of the alkyl ether chain, particularly toward the propyl residue, leads to exclusive oxidation via pathway A. Assuming that the oxidation occurs primarily at the most activated or weakest C–H bond, the site of the oxidation can be shifted by modifying the alkyl side chain used. Specifically, the oxidation site of benzyl ethers was moved away from the more activated benzylic position (**7a** vs **7c**). A similar trend was observed for **2a** vs **2c**. These results open the possibility to use a rather unusual PG, the propyl-residue, in synthetic chemistry.

Regarding the optimization of reaction conditions to promote the formation of target alcohols, these findings clearly suggest that reactions with substrates like **2a**, **4a**, and **7a** cannot be optimized since pathway B is preferred. The conversion of these substrates proceeds rapidly and are typically completed within minutes after the addition of H_2_O_2_ (Figure 2A). To reduce the overall reaction time, we decreased the initial 30 min interval for H_2_O_2_ addition to 3 min. For substrate **2a**, 27% of 2-hexanone and 15% of 2-hexanol were obtained after just 15 min reaction time (Figure 4). Although a similar product distribution to the initial screening was observed, the reaction time was significantly reduced from 4 h to 15 min. In reactions with substrate **4a**, an improved yield of cyclohexanone was detected under optimized conditions, increasing from 41% after 4 h to 55% after 15 min, according to calibrated GC-FID. This makes the reactions catalyzed by UPO23 more practical and less time-consuming.

**Figure 4:**
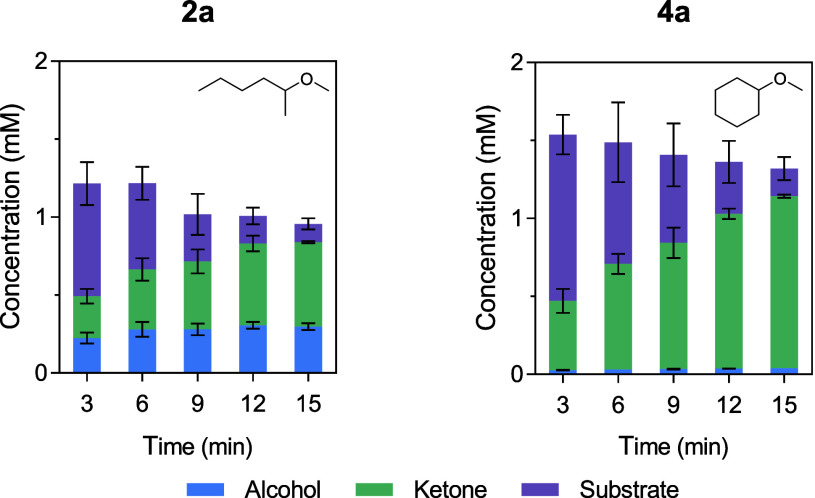
Optimized *O*-dealkylations catalyzed by UPO23. Reactions were performed at 30 °C with shaking in 100 mM tricine buffer (pH 7.5), containing 2 mM substrate and 0.1 mg mL^–1^ lyophilized UPO23. H_2_O_2_ was added up to 5 mM in 3 min intervals. Samples were analyzed by calibrated GC-FID as described below. Concentrations of reaction products are shown as mean values ± SD from reactions replicates (*n* = 3).

Lastly, we performed preparative scale reactions with 50 mg of substrates **7a–c**, a common standard scale in synthetic chemistry, to demonstrate the application of our enzymatic deprotection concept. Based on the findings of this study, we concluded that adding stoichiometric amounts of H_2_O_2_ and no ascorbic acid favors higher yields of the desired benzyl alcohol and reduces the formation of overoxidized products. Since the reaction proceeded rapidly, it was also possible to shorten the H_2_O_2_ addition intervals. The reactions were initiated with 1 mM H_2_O_2_ and quenched after 40 min. Reactions with substrate **7a** yielded a mixture of benzoic acid, benzaldehyde, starting material, and traces of benzyl alcohol. Due to low conversions and poor selectivity, no purification was performed (data not shown). In contrast, substrate **7b** yielded 16.9 mg benzyl alcohol (43% yield) with only small amounts of benzoic acid detected (2 mg, 5% yield). The products were isolated through acid–base extraction; further purification was not needed (Figure S3). Substrate **7c** was converted to benzyl alcohol exclusively (33 mg, 92% yield) (Figure S4). This makes the enzymatic cleavage of a propyl-PG from benzyl alcohol highly suitable for synthetic applications. The results of the preparative scale reactions, in terms of yields and selectivity, are consistent with those observed in the initial screening and demonstrate that the upscaling of reactions is straightforward.

## Conclusion

We identified the unspecific peroxygenase UPO23 as a very useful biocatalyst for the mild and scalable deprotection of a broad range of ether-protected alcohols. The converted substrate scaffolds included primary (**1b**), secondary (**2a–c**, **4a**), and sterically challenging tertiary alcohols (**3a–d**), as well as benzyl alcohols (**7a–c**, **9b–c**, **10c**), containing methyl (**a**), ethyl (**b**), propyl (**c**) and allyl (**d**) PGs.

Interestingly, for several substrates, the detected products were not the target alcohols, but the corresponding aldehydes, ketones, or carboxylic acids. We investigated the potential reaction pathways, in order to gain a better understanding for subsequent reaction optimization. We discovered that the alkyl-chain length had an impact on the reaction pathway. In the presence of short alkyl chains, particularly methoxy groups (**2a**, **4a**, **7a**), pathway B is preferred, involving hydroxylation of the α-carbon of the substrate scaffold (Scheme 2). On the other hand, for substrates with longer alkyl chains (**7b**, **7c**), pathway A is favored, which involves the hydroxylation of the α-carbon of the PG. It is important to note that both pathways can also occur with the same substrate. These findings demonstrate the potential of using longer alkyl chains as PGs for highly regioselective oxidation and therefore deprotection of protected alcohols.

While the optimization toward the target alcohol is not possible for substrates that mainly follow reaction pathway B, we were able to improve reaction conditions and reduce reaction times from 4 h to 15 min for substrates **2a** and **4a** facilitating the application of UPOs. Furthermore, we conducted preparative scale reactions under optimized conditions, in which the conversion of **7b** yielded 43% isolated benzyl alcohol and substrate **7c** was converted to benzyl alcohol with a yield of 92%. The excellent yield for the deprotection of **7c** highlights the value and application of UPO23 for enzymatic deprotection strategies in organic synthesis.

In summary, our results show that UPOs can indeed be used for the (regio-)­selective and efficient removal of alkyl PGs from a variety of alcohol compounds. Future screenings of yet undescribed UPOs or their variants are expected to reveal new enzyme variants capable of cleaving currently inaccessible substrates, including molecules with additional functionalities or multiple PGs. Furthermore, enzyme engineering aimed at improving yield and selectivity toward specific substrates has already been successfully demonstrated for several UPOs and can be applied here too.^52,53^ Together, these developments enable a broader application of UPO catalyzed deprotections in organic synthesis.

## Materials and Methods

### Chemicals and Enzymes

Chemicals were obtained from Szabo Scandic, Eurisotop, Fisher Scientific GmbH, BLD Pharmatech GmbH, Carl Roth, or Sigma-Aldrich and were used as received unless stated otherwise. Compounds **1a–d**, **2a–d**, **3a–d**, **5a**, **7c**, **8a–d**, **9a–d**, **10b–d**, and ^13^C-labeled **7a** were synthesized as described in the Supporting Information. All enzymes (UPOs, catalase) used in this work were purchased from Aminoverse B.V. (Nuth, Netherlands). The putative sequence information for UPO23 is provided in the patent by Novak et al.,^54^ and the sequence is also available in the Supporting Information.

### Enzyme Screening with the Purpald Assay

Reactions were performed in 96-microtiter plates in a final volume of 250 μL. The reaction mixtures contained 1–2.5 mg mL^–1^ lyophilized UPO expression supernatant in buffer (100 mM tricine, pH 7.5). To minimize the screening effort, two substrates belonging to the same substrate class were combined in one reaction to a final concentration of 4 mM each (dissolved in acetonitrile; final cosolvent concentration of 4% (v/v)). Reactions were initiated by the addition of 5 μL H_2_O_2_ (50 mM) and incubated at 30 °C with shaking (450 rpm). For a steady supply, 5 μL H_2_O_2_ were added in intervals of 30 min. After 4 h, reactions were quenched with a UPO stop solution (catalase) provided by Aminoverse, following the manufacturer’s instructions.

To detect *O*-demethylation activity, 200 μL of the UPO reactions were added to 50 μL of a freshly prepared Purpald solution (160 mM in 2 M NaOH). The mixture was incubated for 30 min and the absorbance was detected at 550 nm.^43,55^

### Biotransformations (Analytical Scale)

Standard reactions were performed in 250 μL final volume in 2 mL glass vials containing 0.1 mg mL^–1^ lyophilized UPO23 expression supernatant and 2 mM substrate (dissolved in acetonitrile; final cosolvent concentration of 1% (v/v)) in buffer (100 mM tricine, pH 7.5). Ascorbic acid was added in reactions with aromatic substrates at concentrations of 10 mM (**6a–d**, **8a–d**, **9a–d**, and **10a–d**) or 2 mM (**7a–d**). Reactions were initiated by the addition of 5 μL H_2_O_2_ (50 mM) and incubated at 30 °C and 650 rpm. Every 30 min, 5 μL of H_2_O_2_ were added to the reaction through a Hamilton syringe, until a final concentration of 8 mM was reached. After 4 h, reactions were acidified with HCl (2 M), extracted twice with 250 μL ethyl acetate, using 1 mM methyl benzoate as internal standard, and analyzed via GC-FID. Reactions with substrates **7a**, **7b**, and **7c** were quenched with twice the amount of acetonitrile in filter vials and analyzed via HPLC-UV/vis. To evaluate different time points, reactions with a larger volume were set up in 8 mL glass vials and samples were taken at the indicated times. The volume of H_2_O_2_ added was adjusted according to the volume removed due to sampling. All reactions were performed in independent replicates (*n* ≥ 3).

### Analysis of Released Aldehydes Through Derivatization with 2,4-DNPH

Reactions were performed in 100 μL total volume in 2 mL glass vials at 30 °C and 650 rpm. Reaction mixtures contained 2 mM substrate and 0.1 mg mL^–1^ lyophilized UPO23 expression supernatant received from Aminoverse in buffer (100 mM tricine, pH 7.5). Reactions were initiated by the addition of 0.25 mM H_2_O_2_. After 10 min, reactions were quenched with 20 μL 2,4-DNPH (0.1% (w/v) in acetonitrile, acidified with 0.6 M HCl), and incubated for 20 min. In order to precipitate remaining protein, 80 μL acetonitrile were added. Samples were analyzed via HPLC.

### Biotransformations (Preparative Scale)

All reactions were carried out in a 500 mL Erlenmeyer flask. Reaction mixtures contained 2 mM substrate and 0.1 mg mL^–1^ lyophilized UPO23 expression supernatant from Aminoverse in buffer (100 mM tricine, pH 7.5). Reactions were initiated by the addition of 1 mM of H_2_O_2_. Additionally, another 1 mM of H_2_O_2_ was added after 20 min. The reaction was incubated at 30 °C with continuous shaking at 120 rpm.

For substrate **7b**, which yielded a mixture of benzyl alcohol and benzoic acid, the crude reaction mixture was first basified with aqueous NaOH (pH verified with pH indicator paper). The solution was then extracted twice with diethyl ether as before to isolate benzyl alcohol. The aqueous phase was subsequently acidified to pH ∼1 using HCl (pH verified with pH indicator paper). Benzoic acid was recovered after extracting with the same volume of diethyl ether twice. The organic layers from the two extractions were dried over anhydrous Na_2_SO_4_ separately, filtered, and concentrated under reduced pressure to afford the respective products without further purification. For substrate **7c**, which yielded benzyl alcohol as the sole product, the crude reaction mixture was extracted twice with the same volume of ethyl acetate. The combined organic layers were dried over anhydrous Na_2_SO_4_, filtered, and concentrated under reduced pressure to afford the product without further purification.

### Biotransformation with ^13^C-Labeled Substrates

Reactions were set up in 8 mL glass vials and stirred with a magnetic stirrer at 30 °C. The total volume of the reaction was 800 μL with 2 mM ^13^C-labeled **7a**, 0.1 mg mL^–1^ lyophilized UPO23 expression supernatant and 2 mM H_2_O_2_ in buffer (100 mM tricine, pH 7.5). H_2_O_2_ was added in two steps with 1 mM to initiate the reaction and after 10 min. After 20 min of total reaction time, 50 μL of D_2_O was added and the sample was submitted to ^13^C NMR analysis.

### Chromatographic Analyses

GC-FID analysis was performed on a GC-2010 Plus system (Shimadzu, Duisburg, Germany) equipped with a Zebron ZB-5MSi column (*L* = 30 m; ID = 0.25 mm; FT = 0.25 μm; Phenomenex, Torrance, USA). The flame ionization detector (Shimadzu, Duisburg, Germany) and the injection temperature were both set to a temperature of 320 °C.

The column temperature gradient was set depending on the samples being analyzed. For reactions with substrates **1b**, **2a–c**, and **3a–d**, the initial temperature of 50 °C was held for 5 min, increased to 200 °C at a rate of 20 °C min^–1^ and then further increased to a final temperature of 300 °C at a rate of 30 °C min^–1^ and held for 1.17 min. The column flow was 1.36 mL min^–1^. For reactions with substrate **4a**, the initial temperature of 70 °C was held for 3 min and increased at a rate of 20 °C min^–1^ until 250 °C and held for 5 min. The column flow was 1.3 mL min^–1^. For reactions with substrates **9a**, **9d**, **10a**, and **10d**, the initial temperature of 100 °C was held for 1 min, increased to 190 °C at a rate of 10 °C min^–1^, and then further increased until 250 °C at a rate of 20 °C min^–1^. This temperature was held for 1 min. The column flow was 1.21 mL min^–1^. For reactions with substrates **9b** and **10b**, the initial temperature of 100 °C was held for 1 min. The temperature was then increased to 120 °C at a rate of 10 °C min^–1^ and held for 10 min. It was then further increased to 220 °C at a rate of 20 °C min^–1^ and held for 1 min. The column flow was 1.21 mL min^–1^. For substrates **9c** and **10c**, the initial temperature of 100 °C was held for 1 min and then increased to 120 °C at a rate of 10 °C min^–1^, held for 10 min, then further increased to 160 °C at a rate of 10 °C min^–1^. Lastly, it was increased to 220 °C at a rate of 20 °C min^–1^ until 220 °C and held for 1 min. The column flow was 1.21 mL min^–1^.

Substrate, product, and internal standard peaks were integrated using Shimadzu’s GCMSsolution software and quantified using standard curves based on response factors (relative peak area normalized by the internal standard area). Linear regression parameters are summarized in Table S1 and representative calibration curves shown in Figure S5.

For HPLC analysis, aiming at the quantification of aldehydes or ketones as their 2,4-dinitrophenylhydrazone derivatives, ultrahigh-performance liquid chromatography (uHPLC) was performed, using a 1260 Infinity II series device with a UV/vis diode array detector (Agilent Technologies, Santa Clara, USA). A Luna Omega Polar C18 column (particle size = 5 μm, L = 150 mm, ID = 4.6 mm, Phenomenex, Torrance, USA) was used at 800 bar. The sample volume was 5 μL, column temperature 30 °C, and the flow rate 1 mL min^–1^. The 2,4-dinitrophenylhydrazones were detected by measuring absorbance at 360 nm. Mobile phase A was aqueous 0.1% (v/v) formic acid and mobile phase B was 100% acetonitrile. The HPLC gradient started at 30% B for 5 min, followed by a linear gradient for 25 min to 100% B, decreasing to 30% B in 1 min, and holding 30% B for 7 min. Data were analyzed using OpenLAB CDS 2.4 software (Agilent Technologies, Santa Clara, USA).

The HPLC-UV/vis analysis of substates **7a–d** and the corresponding products was performed on a Nexera LC-40 XR HPLC system (Shimadzu, Kyoto, Japan) comprised of LC-40D XR pumps, a SIL-40C XR autosampler, CTO-40C column oven and a DGU-405 degasser module. Detection was accomplished by an SPD-M40 photo diode. Separations were performed using a XSelect CSH C18 XP column (particle size = 3.5 μm, L = 50 mm, ID = 3.0 mm, Waters, Milford, USA) at 40 °C, a flow rate of 1.3 mL min^–1^ and with acetonitrile and uHPLC grade water containing 0.1% (v/v) formic acid as the mobile phase. Substrate and product peaks were integrated using Shimadzu’s LabSolutions software and quantified using standard curves based on peak areas. Linear regression parameters are summarized in Table S1.

### NMR Analysis

For the preparative scale experiments and the analysis of the synthesized substrates, an Avance UltraShield 400 spectrometer (Bruker Biospin AG, Fällanden, Switzerland) was used. The spectra were recorded in CDCl_3_ solutions and the chemical shift was calibrated to the solvent residual peak.

Labeling experiments were conducted on a Bruker Avance III 600 MHz spectrometer, equipped with a prodigy-cryo BBFO probe. Spectra were directly recorded of the reaction mixture upon addition of D_2_O, applying an inverse-gated decoupling sequence. Further, shifts are referenced to the residual acetonitrile signal at 1.47 ppm.

## Supplementary Material



## Notes

Supporting Information Figures S1–S4, putative sequence of UPO23, details on substrate synthesis, and linear regression parameters (PDF)

## Abbreviations

CYP: P450 monooxygenase
NADPH: reduced nicotinamide adenine dinucleotide phosphate
NMR: nuclear magnetic resonance
PG: protecting group
TLC: thin layer chromatography
uHPLC: ultrahigh-performance liquid chromatography
UPO: unspecific peroxygenase.
