# Supplementary material for: Unspecific Peroxygenases for the Enzymatic Removal of Alkyl Protecting Groups in Organic Synthesis
Source: ACS Catal. 2025 Sep 26;15(20):17090–100. doi: 10.1021/acscatal.5c06385 (PMC12538550; doi:10.1021/acscatal.5c06385)
Supplement: Supplementary file 1 [file cs5c06385_si_001.pdf]

Supporting Information

Unspecific Peroxygenases for the Enzymatic Removal of Alkyl Protecting Groups in Organic Synthesis

Lina A. Csechala,[a] Maximilian Wutscher,[b] Verena Scheibelreiter,[b] Stefan Giparakis,[b] Ina Menyes,[a] Thomas Bayer,[a] Christian Stanetty,[b] Florian Rudroff,[b] Uwe T. Bornscheuer[a]**

[a] Department of Biotechnology & Enzyme Catalysis, Institute of Biochemistry, University of Greifswald, Felix-Hausdorff-Str. 4, 17489 Greifswald, Germany

[b] Institute of Applied Synthetic Chemistry, TU Wien

Getreidemarkt 9, 1060 Vienna, Austria

* Email: uwe.bornscheuer@uni-greifswald.de

* Email: florian.rudroff@tuwien.ac.at

Table of contents

Supplementary Figures 3

Figure S1. Formaldehyde detection by the Purpald assay.. 3

Figure S2. Full uHPLC-chromatograms of reactions derivatized with 2,4-DNPH. 4

Figure S3. NMR spectra of preparative biotransformation of 7b. 5

Figure S4. NMR spectra of preparative biotransformation of 7c. 6

Sequence of UPO23 7

Synthetic Procedures 7

General procedure A for the synthesis of substrates 8a–d 7

General procedure B for the synthesis of substrates 9b–d 7

General procedure C for the synthesis of substrates 1a, 2a, and 3a 7

General procedure D for the synthesis of 1b–d, 2b–d, 3b–d, 5a, 7a, and 10b–d 8

Procedure for the synthesis of substrate 9a 8

^{1}H-NMR analysis 9

Linear Regression Parameters 15

Table S1. Linear regression parameters of standard curves used for compound quantification 15

Figure S5. Representative calibration curves. 16

References 17

Supplementary Figures

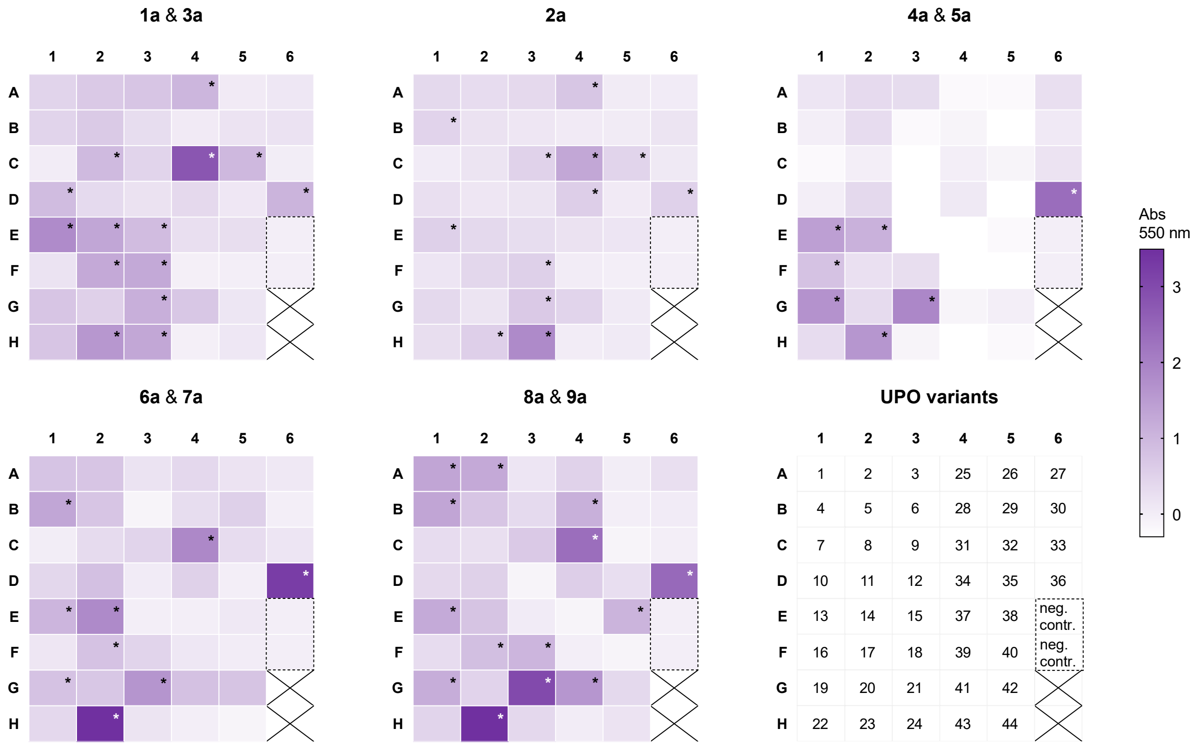

Figure S1. Formaldehyde detection by the Purpald assay. Reactions were performed at 30 °C with shaking for 4 h in 100 mM tricine buffer (pH 7.5), containing two combined substrates (2 mM each) and 1-2.5 mg·mL^{-1} lyophilized UPO23. H_2O_2 was added in 30 min intervals (up to 8 mM). For aldehyde detection 200 µL of the UPO reaction solution was mixed with 50 µL of a Purpald solution (160 mM in 2 M NaOH) and incubated at 25 °C for 20 min. Absorbance was measured at 550 nm, with negative controls used as blanks. Reactions with absorbance values corresponding to the top 25% are indicated with an asterisk. UPO23 was located in cell H2.

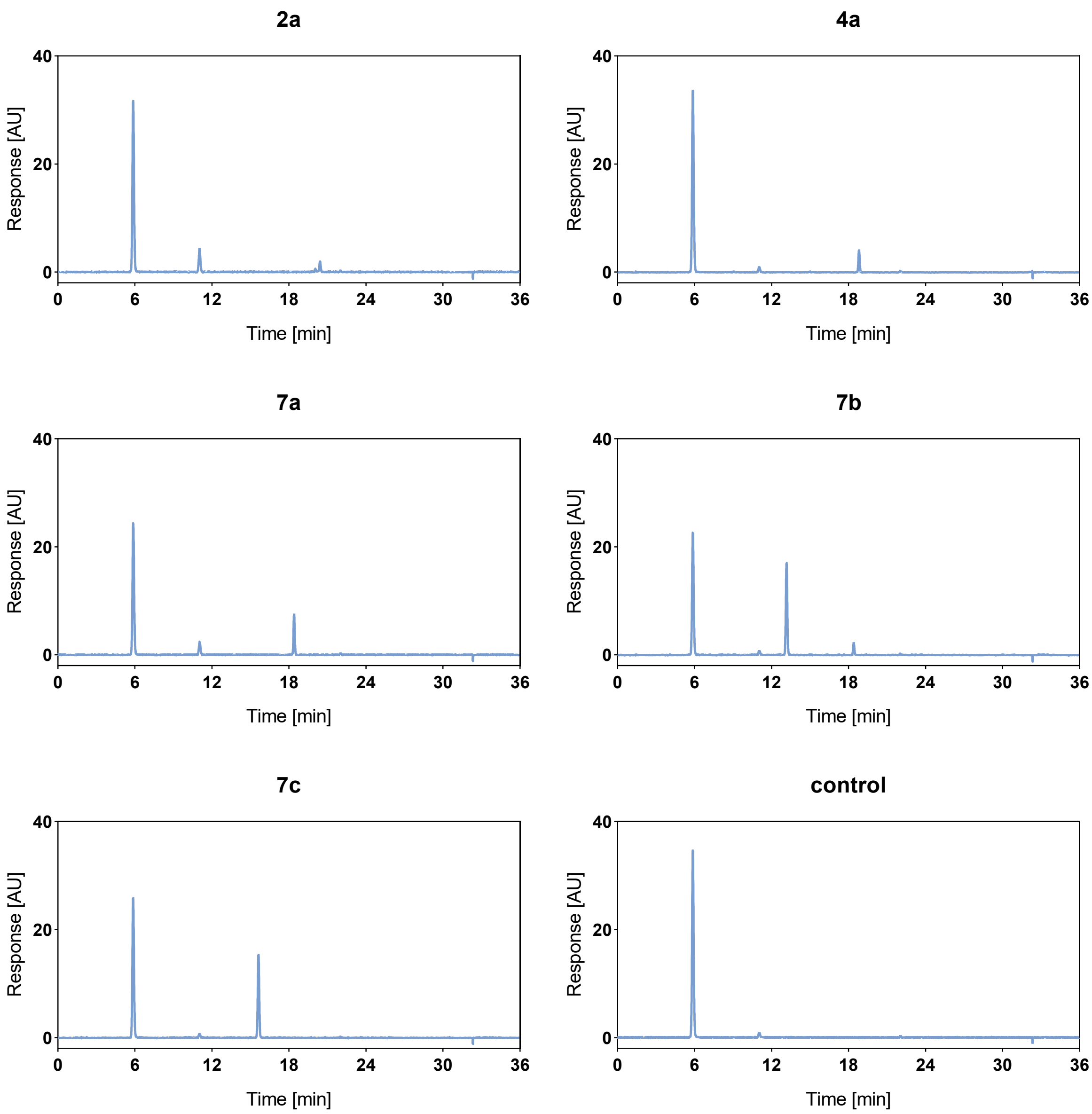

Figure S2. Full uHPLC-chromatograms of reactions derivatized with 2,4-DNPH. Reactions were performed at 30 °C with shaking at 650 rpm for 10 min in 100 mM tricine buffer (pH 7.5), 2 mM substrate and 0.1 mg·mL^{-1} lyophilized UPO23. The reaction was initiated with 0.25 mM H_2O_2 and after 10 min quenched and derivatized with 2,4-DNPH. The control did not contain substrate. Reactions were performed in triplicates with exemplary chromatograms shown. 5.9 min: 2,4-DNPH, 11 min: formaldehyde-2,4-DNPH, 13.2 min: acetaldehyde-2,4-DNPH, 15.6 min: propionaldehyde-2,4-DNPH, 18.4 min: benzaldehyde-2,4-DNPH, 18.8 min: cyclohexanone-2,4-DNPH, 20 min: 2-hexanone-2,4-DNPH.

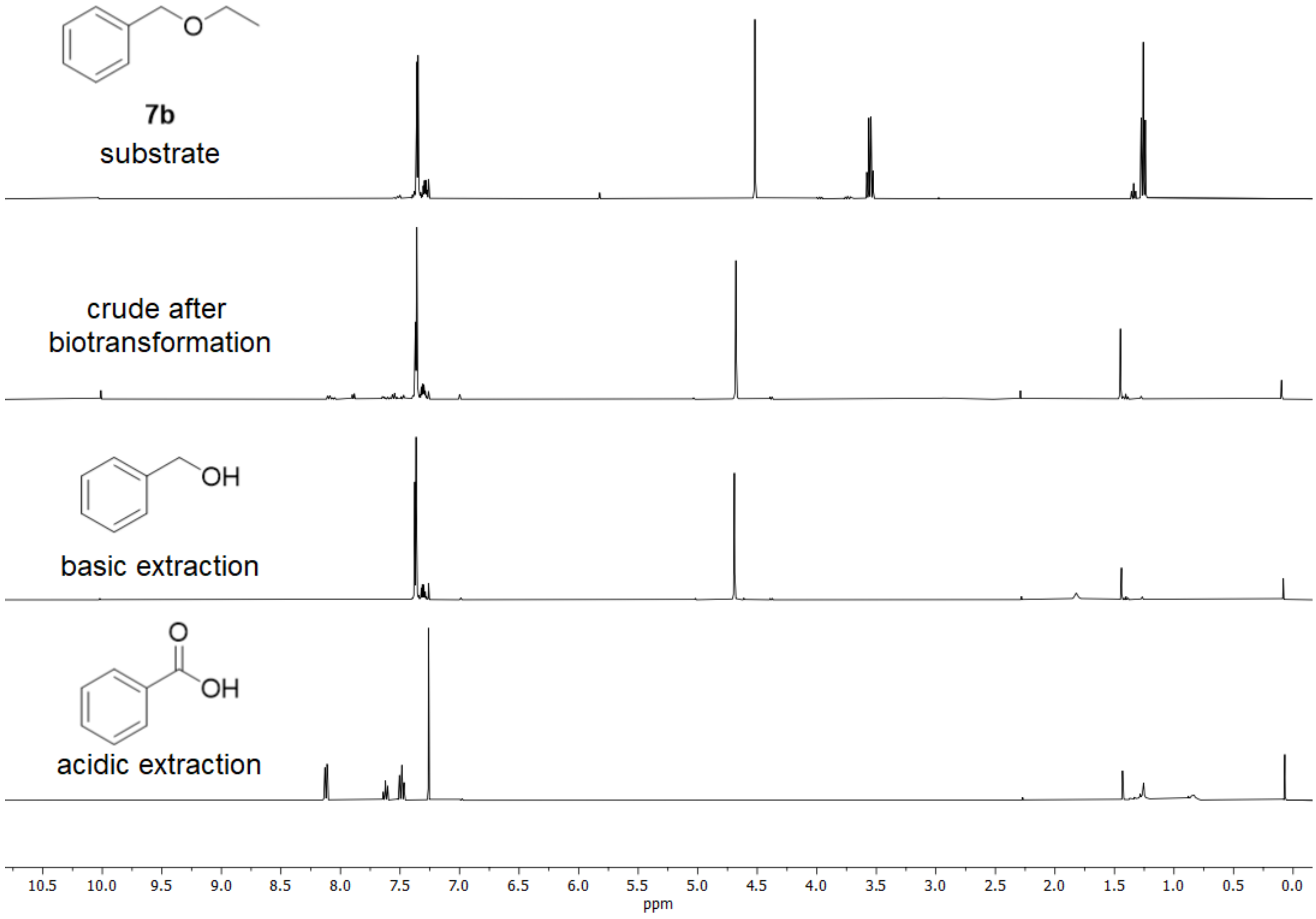

Figure S3. NMR spectra of preparative biotransformation of **7b**. From top to bottom: substrate **7b**, crude extract after biotransformation, extract from basic aqueous phase containing benzyl alcohol, extract from acidic aqueous phase containing benzoic acid.

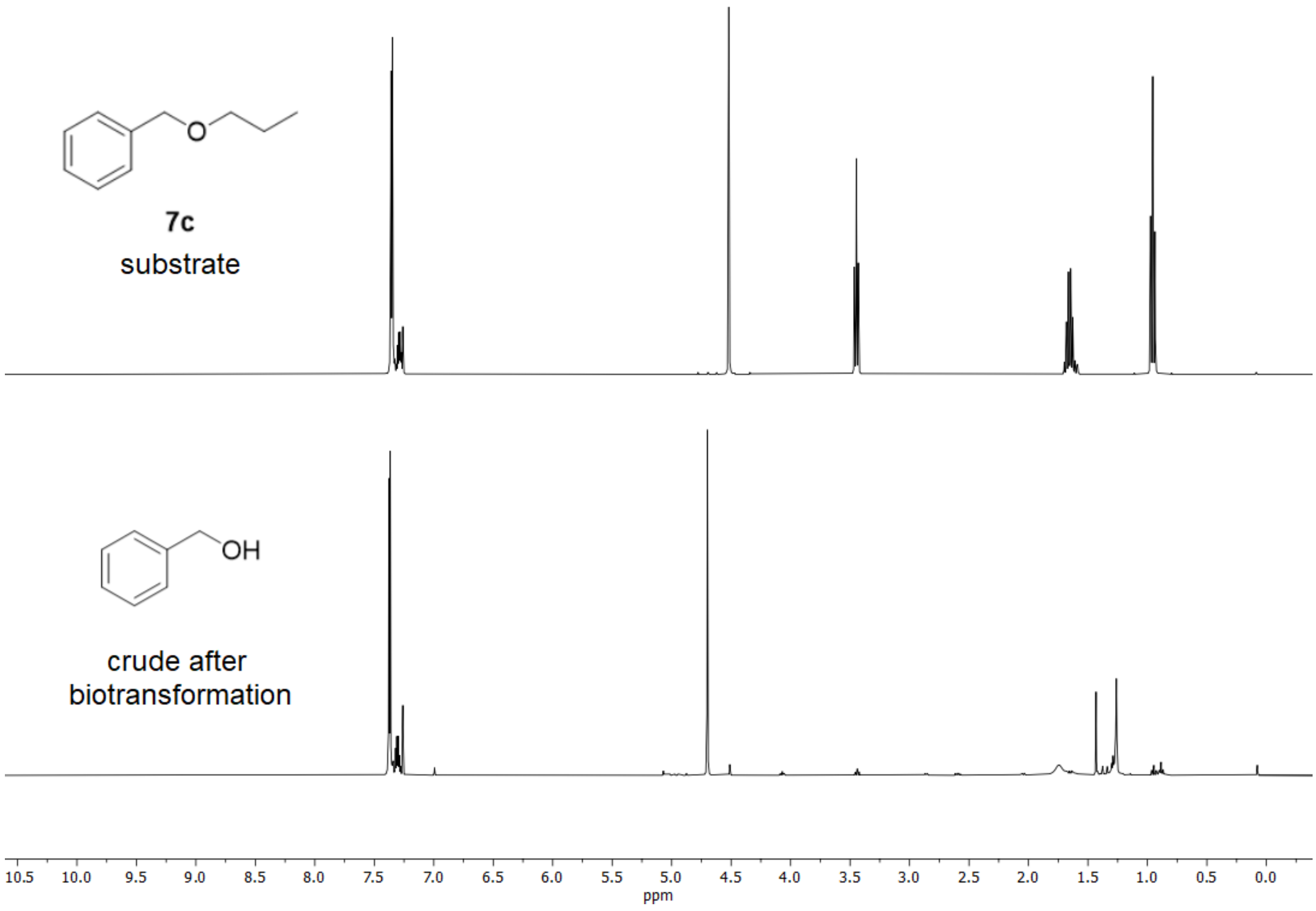

Figure S4. NMR spectra of preparative biotransformation of **7c**. From top to bottom: substrate **7c**, crude extract after biotransformation.

Sequence of UPO23

The putative sequence of UPO23 according to the patent, is as follows:[1]

MKTATLLFLAAGLTQTQAFPSQGAAPHPLPWSPPGPNDVRAPCPMLNTLANHGYLP
HNGKNITEQHTINALYNALGIDAELSAFLHQEAVTTNPTPNATTFSLNDLSRHDILEHD
ASLSRQDAYFGDNHDFNQTIFDETRSYWTSPIIDVKQAALSRQARVNTSMATNPNYT
MSELGASFSYGETAAYIIVLGDKENGLVNRSRVEYLFENERLPLDLGWTRAKENITFDD
LRTMLNRIVNATGGESEFDRELAKRGGVHVGRWRGY

Synthetic Procedures

General procedure A for the synthesis of substrates 8a–d

Substituted benzyl alcohol (1.0 equiv.) and K_2CO_3 (3.0 equiv.) were dissolved in DMF (0.1 M). Then, the corresponding alkyl halogenide (1.1 equiv.) was added and the suspension was stirred overnight at room temperature. The reaction mixture was diluted with ethyl acetate and washed with water 3 times and brine twice. The organic layers were dried over $MgSO_4$, filtered and the solvent was evaporated under reduced pressure. Purification was performed by column chromatography over silica.[2]

General procedure B for the synthesis of substrates 9b–d

A mixture of $Yb(OTf)_3$ (0.01 equiv.), substituted benzyl alcohol (1 equiv.) and the corresponding alkyl alcohol (5 equiv.) in acetonitrile (0.1 M) was stirred at 80 °C for 5 h. After dilution with water, the mixture was extracted with ethyl acetate. The organic layers were dried over $MgSO_4$, filtered and the solvent was evaporated under reduced pressure. Purification was performed by column chromatography over silica.[3]

General procedure C for the synthesis of substrates 1a, 2a, and 3a

A flame-dried 25 mL screw cap vial equipped with septum and magnetic stirring was charged with NaH (1.5 equiv.) and suspended with DMA (0.1 M). Subsequently, the alcohol (1.0 equiv.) was added dropwise to the mixture over a period of 30 min. Methyl iodide (1.25 equiv.) was then added

and the resulting mixture was stirred at room temperature for 16 h. Reaction progress was monitored via TLC (PE / EtOAc 2:1). Upon complete consumption of the starting material, solid ammonium chloride was added. The desired product was subsequently isolated by direct distillation from the solution at 80 °C and 100 mbar, yielding a colorless oil.

General procedure D for the synthesis of 1b–d, 2b–d, 3b–d, 5a, 7a, and 10b–d

Alcohol (1.0 equiv.) and NaH (2.0 equiv. for single alkylation, 4.0 equiv. for double alkylation) were dissolved in DMF (0.5M). Then, the corresponding alkyl halogenide (2.0 equiv. for single alkylation, 4.0 equiv. for double alkylation) was added and the suspension was stirred overnight at room temperature. The reaction mixture was diluted with ethyl acetate and washed with water three times and brine twice. The organic layers were dried over $MgSO_4$, filtered and the solvent was evaporated under reduced pressure.[2]

Procedure for the synthesis of substrate 9a

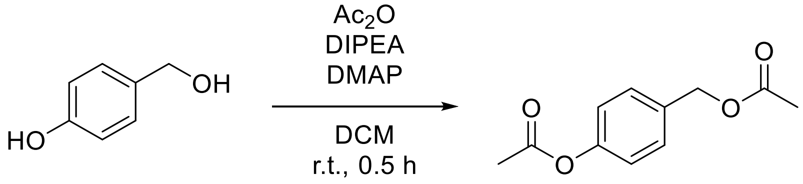

A flame dried Schlenk-flask, equipped with septum and magnetic stirring, was charged with 4-hydroxy benzyl alcohol (1.24 g, 10.0 mmol, 1.0 equiv.) and DMPA (100 mg, 0.8 mmol, 0.08 equiv.). The vessel was then purged with argon utilizing standard Schlenk techniques. Then, 50 mL of dry DCM was added followed by the dropwise addition of Ac2O (4.1 mL, 44.0 mmol, 4.4 equiv.). Subsequently DIPEA (3.6 mL, 21.6 mmol, 2.16 equiv.) was added dropwise over a period of 10 min. Reaction progress was monitored via TLC (PE / EtOAc 1:1). After 30 min full consumption of the starting material was observed. The mixture was then transferred into a separation funnel and washed with H_2O (25 mL), 2 N HCl (25 mL) and Na_2CO_3 (15 mL). The organic phase was then dried over Na_2SO_4 and the solvent removed under reduced pressure. The crude was further purified via filtration over a pad of silica (PE / EtOAc 3:1) resulting in a colorless oil (1.81 g, 8.7 mmol, 87 %).

^{1}H-NMR (400 MHz, Chloroform-d): δ 7.41 – 7.33 (m, 2H), 7.12 – 7.04 (m, 2H), 5.09 (s, 2H), 2.30 (s, 3H), 2.10 (s, 3H).

1H-NMR Spectrum according to literature.[4]

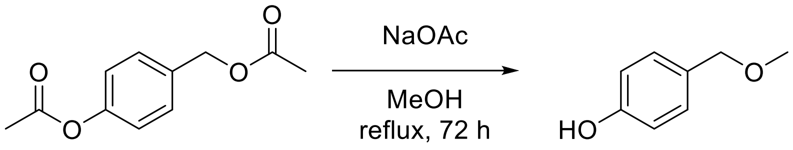

9a

A flame-dried three necked round bottom flask, equipped with magnetic stirring, septum and condenser, was charged with 4-(acetyloxy)benzyl acetate (0.80 g, 3.8 mmol, 1.0 equiv.). The vessel was purged with Argon utilizing standard Schlenk techniques. 10 mL of MeOH was then added followed by NaOAc (0.31 g, 3.8 mmol, 1.0 equiv.) under Argon counter flow. The reaction progress was monitored via TLC (PE / EtOAc 2:1). The reaction was refluxed for 72 h. Afterwards, the heating source was removed allowing the mixture to cool to room temperature. Water was then added and the mixture extracted with EtOAc. The combined organics were dried over Na_2SO_4, filtered and the solvent removed under reduced pressure. The crude was then further purified via column chromatography (PE /EtOAc 1:1) leading to a white crystalline solid (0.50 g, 3.6 mmol, 95 %)

^{1}H-NMR (400 MHz, Chloroform-*d*) δ: 7.23 – 7.15 (m, 2H), 6.79 – 6.71 (m, 2H), 5.84 (s, 1H), 4.40 (s, 2H), 3.38 (s, 3H).

^{1}H-NMR Spectrum according to literature.[5]

^{1}H-NMR analysis

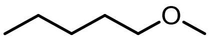

1a

^{1}H-NMR (400 MHz, Chloroform-*d*) δ 3.36 (t, J = 6.7 Hz, 2H), 3.33 (s, 3H), 1.64 – 1.51 (m, 2H), 1.40 – 1.25 (m, 4H), 0.96 – 0.84 (m, 3H).

^{1}H-NMR Spectrum according to literature.[6]

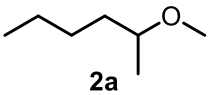

2a

^{1}H-NMR (400 MHz, Chloroform-*d*) δ 3.31 (s, 3H), 3.32 – 3.21 (m, 1H), 1.56 – 1.48 (m, 1H), 1.43 – 1.23 (m, 5H), 1.12 (d, J = 6.1 Hz, 3H), 0.94 – 0.86 (m, 3H).

^{1}H-NMR Spectrum according to literature.[7]

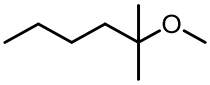

3a

^{1}H-NMR (400 MHz, Chloroform-*d*) δ 3.17 (s, 3H), 1.49 – 1.40 (m, 2H), 1.37 – 1.22 (m, 4H), 1.13 (s, 6H), 0.95 – 0.86 (m, 3H).

^{1}H-NMR Spectrum according to literature.[8]

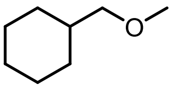

5a

^{1}H-NMR (400 MHz, Chloroform-*d*) δ 3.32 (s, 3H, CH_3), 3.17 (d, *J* = 6.48 Hz, 2H, CH_2), 1.80 – 1.63 (m, 5H), 1.62 – 1.52 (m, 1H), 1.33 – 1.10 (m, 3H), 0.99 – 0.81 (m, 2H).

^{1}H-NMR Spectrum according to literature.[9]

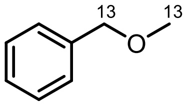

partially ^{13}C labelled **7a**

^{1}H-NMR (400 MHz, Chloroform-*d*) δ 7.40 – 7.28 (m, 5H), 4.47 (dd, *J* = 141.1, 4.2 Hz, 2H), 3.40 (dd, *J* = 141.0, 5.2 Hz, 3H).

^{13}C-NMR (101 MHz, Chloroform-*d*) δ 138.24 (dd, *J* = 48.3, 2.8 Hz), 128.53 (d, *J* = 3.8 Hz), 127.87 (d, *J* = 3.1 Hz), 127.78 (s), 74.85 (d, *J* = 1.8 Hz), 58.24 (d, *J* = 1.8 Hz).

^{1}H-NMR Spectrum according to literature.[10]

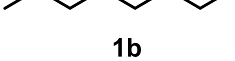

1b

^{1}H-NMR (400 MHz, Chloroform-*d*) δ 3.46 (q, *J* = 7.0 Hz, 2H), 3.40 (t, *J* = 6.8 Hz, 2H), 1.62 – 1.51 (m, 2H), 1.38 – 1.27 (m, 4H), 1.19 (t, *J* = 7.0 Hz, 3H), 0.95 – 0.84 (m, 3H).

^{1}H-NMR Spectrum according to literature.[11]

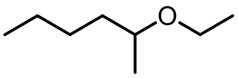

2b

^{1}H-NMR (400 MHz, Chloroform-*d*) δ 3.53 (dq, *J* = 9.2, 7.0 Hz, 1H), 3.47 – 3.28 (m, 2H), 1.57 – 1.46 (m, 1H), 1.41 – 1.22 (m, 5H), 1.18 (t, *J* = 7.0 Hz, 3H), 1.12 (d, *J* = 6.1 Hz, 3H), 0.93 – 0.85 (m, 3H).

^{1}H-NMR Spectrum according to literature.[12]

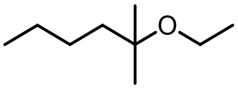

3b

^{1}H-NMR (400 MHz, Chloroform-*d*) δ 3.36 (q, *J* = 7.0 Hz, 2H), 1.49 – 1.40 (m, 2H), 1.35 – 1.22 (m, 4H), 1.15 (t, *J* = 7.0 Hz, 3H), 1.13 (s, 6H), 0.95 – 0.84 (m, 3H).

^{13}C-NMR (151 MHz, Chloroform-*d*) δ 74.6, 56.4, 40.0, 26.3, 25.9, 23.4, 16.4, 14.3.

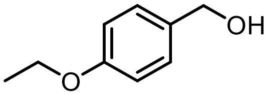

8b

^{1}H-NMR (400 MHz, Chloroform-*d*) δ 7.24 – 7.16 (m, 2H), 6.85 – 6.76 (m, 2H), 4.53 (s, 2H), 3.96 (q, *J* = 7.0 Hz, 2H), 1.34 (t, *J* = 7.0 Hz, 3H).

^{1}H-NMR Spectrum according to literature.[13]

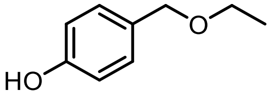

9b

^{1}H-NMR (400 MHz, DMSO-*d*) δ 9.33 (s, 1H), 7.14 – 7.06 (m, 2H), 6.75 – 6.67 (m, 2H), 4.30 (s, 2H), 3.41 (q, *J* = 7.0 Hz, 2H), 1.11 (t, *J* = 7.0 Hz, 3H).

^{1}H-NMR Spectrum according to literature.[14]

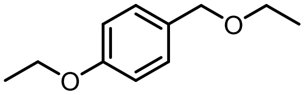

10b

^{1}H-NMR (400 MHz, Chloroform-*d*) δ 7.30 – 7.21 (m, 2H), 6.91 – 6.83 (m, 2H), 4.43 (s, 2H), 4.03 (q, *J* = 7.0 Hz, 2H), 3.51 (q, *J* = 7.0 Hz, 2H), 1.41 (t, *J* = 7.0 Hz, 3H), 1.23 (t, *J* = 7.0 Hz, 3H).

^{13}C-NMR (151 MHz, Chloroform-*d*) δ 158.6, 130.7, 129.4, 114.5, 72.6, 65.6, 63.6, 15.4, 15.0.

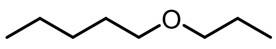

1c

^{1}H-NMR (400 MHz, Chloroform-*d*) δ 3.38 (dt, J = 14.5, 6.8 Hz, 4H), 1.65 – 1.52 (m, 4H), 1.38 – 1.28 (m, 4H), 0.92 (t, J = 7.3 Hz, 6H).

^{13}C-NMR (101 MHz, Chloroform-*d*) δ 72.7, 71.1, 29.6, 28.5, 23.1, 22.7, 14.2, 10.7.

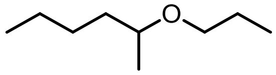

2c

^{1}H-NMR (400 MHz, Chloroform-*d*) δ 3.48 – 3.24 (m, 3H), 1.63 – 1.45 (m, 3H), 1.43 – 1.16 (m, 3H), 1.11 (d, J = 6.1 Hz, 3H), 0.95 – 0.85 (m, 6H).

^{13}C-NMR (151 MHz, Chloroform-*d*) δ 75.5, 70.3, 36.6, 28.0, 23.5, 23.0, 19.9, 14.2, 10.8.

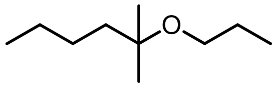

3c

^{1}H-NMR (400 MHz, Chloroform-*d*) δ 3.25 (t, J = 6.8 Hz, 2H), 1.62 – 1.40 (m, 4H), 1.36 – 1.21 (m, 4H), 1.13 (s, 6H), 0.90 (t, J = 7.4 Hz, 6H).

^{13}C-NMR (151 MHz, Chloroform-d) δ 74.4, 62.9, 40.2, 26.3, 25.8, 24.0, 23.5, 14.3, 10.9.

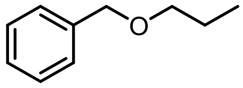

7c

^{1}H-NMR (400 MHz, Chloroform-*d*) δ 7.35 (d, J = 4.4 Hz, 4H), 7.32 – 7.27 (m, 1H), 4.52 (s, 2H), 3.45 (t, J = 6.7 Hz, 2H), 1.65 (h, J = 7.1 Hz, 2H), 0.96 (t, J = 7.4 Hz, 3H).

^{1}H-NMR Spectrum according to literature.[15]

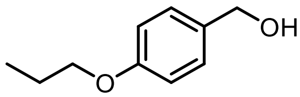

8c

^{1}H-NMR (400 MHz, Chloroform-*d*) δ 7.31 – 7.24 (m, 2H), 6.92 – 6.85 (m, 2H), 4.61 (s, 2H), 3.92 (t, J = 6.6 Hz, 2H), 1.81 (dtd, J = 13.9, 7.4, 6.6 Hz, 2H), 1.04 (t, J = 7.4 Hz, 3H).

^{1}H-NMR Spectrum according to literature.[16]

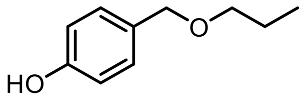

9c

^{1}H-NMR (400 MHz, DMSO-*d*) δ 9.33 (s, 1H), 7.14 – 7.06 (m, 2H), 6.75 – 6.69 (m, 2H), 4.30 (s, 2H), 3.32 (t, *J* = 6.8 Hz, 4H), 1.51 (dtd, *J* = 13.9, 7.4, 6.5 Hz, 2H), 0.86 (t, *J* = 7.4 Hz, 3H).

^{13}C-NMR (151 MHz, DMSO-d_6) δ 157.6, 134.4, 127.9, 114.0, 68.9, 62.6, 22.1, 10.4.

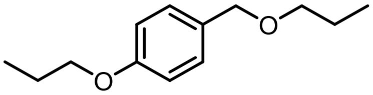

10c

^{1}H-NMR (400 MHz, Chloroform-*d*) δ 7.29 – 7.21 (m, 2H), 6.91 – 6.83 (m, 2H), 4.44 (s, 2H), 3.92 (t, *J* = 6.6 Hz, 2H), 3.41 (t, *J* = 6.7 Hz, 2H), 1.81 (dtd, *J* = 13.9, 7.4, 6.6 Hz, 2H), 1.69 – 1.56 (m, 2H), 1.04 (t, *J* = 7.4 Hz, 3H), 0.93 (t, *J* = 7.4 Hz, 3H).

^{13}C-NMR (151 MHz, Chloroform-*d*) δ 158.8, 130.8, 129.3, 114.5, 72.7, 72.0, 69.7, 23.1, 22.7, 10.8, 10.7.

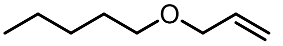

1d

^{1}H-NMR (400 MHz, Chloroform-*d*) δ 5.92 (ddt, *J* = 17.2, 10.4, 5.6 Hz, 1H), 5.27 (dq, *J* = 17.2, 1.7 Hz, 1H), 5.16 (dq, *J* = 10.4, 1.3 Hz, 1H), 3.96 (dt, *J* = 5.6, 1.4 Hz, 2H), 3.42 (t, *J* = 6.7 Hz, 2H), 1.65 – 1.54 (m, 2H), 1.39 – 1.29 (m, 4H), 0.94 – 0.86 (m, 3H).

^{13}C-NMR (151 MHz, Chloroform-*d*) δ 135.3, 116.8, 71.9, 70.6, 29.6, 28.5, 22.7, 14.2.

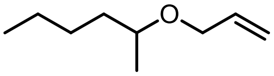

2d

^{1}H-NMR (400 MHz, Chloroform-*d*) δ 5.92 (ddt, *J* = 17.2, 10.3, 5.6 Hz, 1H), 5.31 – 5.21 (m, 1H), 5.19 – 5.09 (m, 1H), 4.02 (ddt, *J* = 12.8, 5.5, 1.5 Hz, 1H), 3.92 (ddt, *J* = 12.7, 5.7, 1.4 Hz, 1H), 3.48 – 3.36 (m, 1H), 1.65 – 1.48 (m, 2H), 1.46 – 1.20 (m, 4H), 1.13 (d, *J* = 6.1 Hz, 3H), 0.96 – 0.85 (m, 3H).

^{13}C-NMR (151 MHz, Chloroform-*d*) δ 135.8, 116.4, 75.1, 69.5, 36.5, 27.9, 22.9, 19.8, 14.2.

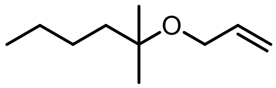

3d

^{1}H-NMR (400 MHz, Chloroform-*d*) δ 5.92 (ddt, *J* = 17.2, 10.6, 5.4 Hz, 1H), 5.31 – 5.22 (m, 1H), 5.10 (dq, *J* = 10.4, 1.6 Hz, 1H), 3.87 (dt, *J* = 5.4, 1.6 Hz, 2H), 1.48 (ddd, *J* = 8.4, 4.8, 2.1 Hz, 2H), 1.36 – 1.23 (m, 4H), 1.16 (s, 7H), 0.96 – 0.86 (m, 3H).

^{13}C-NMR (151 MHz, Chloroform-*d*) δ 136.5, 115.7, 75.2, 62.8, 40.2, 26.3, 25.7, 23.4, 14.3.

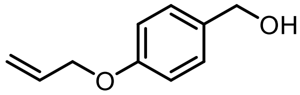

8d

^{1}H-NMR (400 MHz, Chloroform-*d*) δ 7.32 – 7.26 (m, 2H), 6.93 – 6.87 (m, 2H), 6.06 (ddt, *J* = 17.3, 10.6, 5.3 Hz, 1H), 5.41 (dq, *J* = 17.3, 1.6 Hz, 1H), 5.29 (dq, *J* = 10.5, 1.4 Hz, 1H), 4.62 (s, 2H), 4.54 (dt, *J* = 5.3, 1.6 Hz, 2H).

^{1}H-NMR Spectrum according to literature.[17]

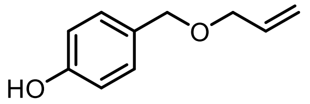

9d

^{1}H-NMR (400 MHz, Chloroform-*d*) δ 7.24 – 7.17 (m, 2H), 6.80 – 6.72 (m, 2H), 5.95 (ddt, *J* = 17.3, 10.3, 5.7 Hz, 1H), 5.30 (dq, *J* = 17.2, 1.6 Hz, 1H), 5.21 (dq, *J* = 10.4, 1.4 Hz, 1H), 4.45 (s, 2H), 4.02 (dt, *J* = 5.8, 1.4 Hz, 2H).

^{1}H-NMR Spectrum according to literature.[18]

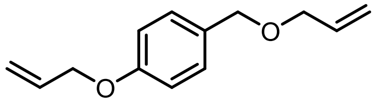

10d

^{1}H-NMR (400 MHz, Chloroform-*d*) δ 7.32 – 7.23 (m, 2H), 6.94 – 6.86 (m, 2H), 6.13 – 5.88 (m, 2H), 5.41 (dq, *J* = 17.3, 1.6 Hz, 1H), 5.35 – 5.27 (m, 1H), 5.31 – 5.24 (m, 1H), 5.20 (dq, *J* = 10.4, 1.4 Hz, 1H), 4.54 (dt, *J* = 5.3, 1.6 Hz, 2H), 4.45 (s, 2H), 4.01 (dt, *J* = 5.7, 1.4 Hz, 2H).

^{13}C-NMR (151 MHz, Chloroform-*d*) δ 158.3, 135.0, 133.4, 130.7, 129.5, 117.8, 117.2, 114.8, 71.9, 71.04, 69.0.

Linear Regression Parameters

Table S1. Linear regression parameters of standard curves used for compound quantification**.** Calibration curves were prepared with 4 or 5 concentrations and analyzed with a linear regression.

Compound	Equation	R square	n
**1b**	Y = 0.4302*X – 0.008939	0.973	3
**1-Pentanol**	Y = 0.5292*X – 0.02378	0.993	3
**2a**	Y = 0.2839*X – 0.006071	0.985	3
**2b**	Y = 0.5499*X – 0.01828	0.943	3
**2-Hexanol**	Y = 0.4965*X – 0.02051	0.997	3
**2-Hexanone**	Y = 0.7019*X – 0.02125	0.990	3
**3a**	Y = 0.5926*X – 0.02909	0.992	3
**3d**	Y = 0.2928*X – 0.005837	0.940	3
**2-Methyl-2-hexanol**	Y = 0.6540*X – 0.01472	0.991	3
**4a**	Y = 0.6963*X – 0.01717	0.994	3
**Cyclohexanol**	Y = 1.060*X – 0.01537	0.998	3
**Cyclohexanone**	Y = 0.8169*X – 0.02081	0.996	3
**7a**	Y = 377495*X	0.963	1
**7b**	Y = 323833*X	0.991	1
**7c**	Y = 258489*X	0.994	1
**Benzyl alcohol**	Y = 388013*X	0.974	1
**Benzaldehyde**	Y = 187398*X	0.993	1
**Benzoic acid**	Y = 485909*X	0.976	1
**9b**	Y = 1.181*X – 0.08591	0.954	3
**9c**	Y = 1.421*X – 0.09092	0.988	3
**9d**	Y = 2.080*X – 0.09143	0.998	3
**4-(Hydroxymethyl)phenol**	Y = 1.219*X – 0.04893	0.966	3
**10c**	Y = 0.2278*X – 0.007485	0.911	3
**(4-Propoxyphenyl)methanol**	Y = 0.5558*X – 0.02568	0.989	3

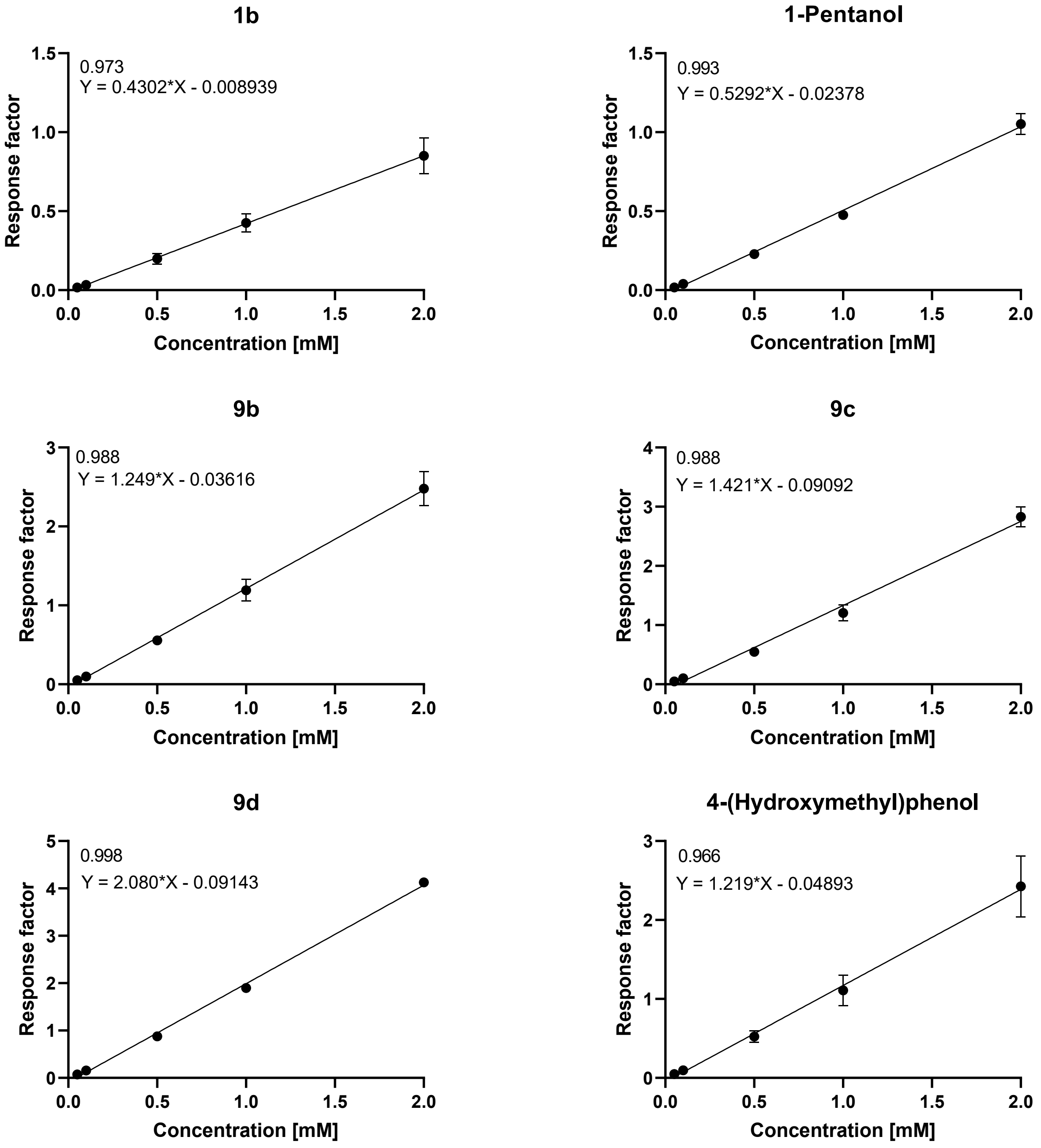

Figure S5. Representative calibration curves. The response factors (relative peak area normalized by the internal standard area) were calculated from three independent replicates. A complete set of linear regression parameters is listed in **Table S1**.

References

(1) Novak, K. D.; Glieder, A.; Weninger, A.; Reisinger, C.; Rinnofner, C.; Pichler, C. Recombinant Heme Thiolate Oxygenases. US20220282227A1, August 8, 2022.

(2) Eleftheriadis, N.; Thee, S.; Te Biesebeek, J.; Van Der Wouden, P.; Baas, B.-J.; Dekker, F. J. Identification of 6-Benzyloxysalicylates as a Novel Class of Inhibitors of 15-Lipoxygenase-1. *Eur. J. Med. Chem.* **2015**, *94*, 265–275. https://doi.org/10.1016/j.ejmech.2015.03.007.

(3) Kawada, A.; Yasuda, K.; Abe, H.; Harayama, T. Rare Earth Metal Trifluoromethanesulfonates Catalyzed Benzyl-Etherification. *Chem. Pharm. Bull.* **2002**, *50* (3), 380–383. https://doi.org/10.1248/cpb.50.380.

(4) Zhou, X.-Y.; Chen, X. Na_2CO_3-Catalyzed *O*-Acylation of Phenols for the Synthesis of Aryl Carboxylates with Use of Alkenyl Carboxylates. *Synlett* **2018**, *29* (17), 2321–2325. https://doi.org/10.1055/s-0037-1610265.

(5) Zhang, S.; Wang, X.; Li, R.; Lou, Z.; Zhan, H.; Yamaguchi, M. Enhanced Ester Dechloroacetylation through Transesterification with Trimethoxyborane. *Org. Biomol. Chem.* **2025**, *23* (17), 4058–4062. https://doi.org/10.1039/D5OB00255A.

(6) McMillan, A. J.; Sieńkowska, M.; Di Lorenzo, P.; Gransbury, G. K.; Chilton, N. F.; Salamone, M.; Ruffoni, A.; Bietti, M.; Leonori, D. Practical and Selective Sp^3 C−H Bond Chlorination via Aminium Radicals. *Angew. Chem. Int. Ed.* **2021**, *60* (13), 7132–7139. https://doi.org/10.1002/anie.202100030.

(7) Han, X.; Nguyen, R.; Ouyang, W.; Hu, M.; Len, C. Reductive *O*-Alkylation of Methanol by Ketones Using a Heterogeneous Palladium Catalyst under Continuous Flow Conditions. *New J. Chem.* **2025**, *49* (3), 779–786. https://doi.org/10.1039/D4NJ03663H.

(8) Seyfert, F.; Mitha, M.; Wagenknecht, H. Nucleophilic Alkoxylations of Unactivated Alkyl Olefins and α-Methyl Styrene by Photoredox Catalysis. *Eur. J. Org. Chem.* **2021**, *2021* (5), 773–776. https://doi.org/10.1002/ejoc.202001533.

(9) Sakoda, K.; Yamaguchi, S.; Mitsudome, T.; Mizugaki, T. Selective Hydrodeoxygenation of Esters to Unsymmetrical Ethers over a Zirconium Oxide-Supported Pt–Mo Catalyst. *JACS Au* **2022**, *2* (3), 665–672. https://doi.org/10.1021/jacsau.1c00535.

(10) Harnedy, J.; Maashi, H. A.; El Gehani, A. A. M. A.; Burns, M.; Morrill, L. C. Deconstructive Functionalization of Unstrained Cycloalkanols via Electrochemically Generated Aromatic Radical Cations. *Org. Lett.* **2023**, *25* (9), 1486–1490. https://doi.org/10.1021/acs.orglett.3c00219.

(11) Kumar, H. M. S.; Joyasawal, S.; Reddy, B. V. S.; Chakravarthy, P. P.; Krishna, A. D.; Yadav, J. S. Reaction of Orthoesters with Alcohols in the Presence of Acidic Catalysts: A Study. *ChemInform* **2005**, *36* (48), chin.200548058. https://doi.org/10.1002/chin.200548058.

(12) Radhakrishnan, S.; Franken, J.; Martens, J. A. Selective Synthesis of 2-Ethoxy Alkanes through Ethoxylation of 1-Alkenes with Bioethanol over Zeolite Beta Catalyst in a Liquid Phase Continuous Process. *Green Chem.* **2012**, *14* (5), 1475. https://doi.org/10.1039/c2gc35220f.

(13) Qi, S.; Liu, K.; Liu, H.; Zhang, G.; Zheng, H.; Sun, J.; Sun, B.; Lou, H. Discovery of Potent Quinone Oxidoreductase 2 Inhibitors to Overcome TRAIL Resistance of Non-Small Cell Lung Cancer. *Eur. J. Med. Chem.* **2025**, *288*, 117382. https://doi.org/10.1016/j.ejmech.2025.117382.

(14) Zhong, L.; Wu, J.; Wu, X. Gallic Acid and EGFR Target Antibody Composition and Application Thereof in Lung Cancer. CN110812479A, February 21, 2020.

(15) Li, L.; Wang, X.; Fu, N. Electrochemical Nickel-Catalyzed Hydrogenation. *Angew. Chem. Int. Ed.* **2024**, *63* (22), e202403475. https://doi.org/10.1002/anie.202403475.

(16) Ichikawa, T.; Netsu, M.; Mizuno, M.; Mizusaki, T.; Takagi, Y.; Sawama, Y.; Monguchi, Y.; Sajiki, H. Development of a Unique Heterogeneous Palladium Catalyst for the Suzuki–Miyaura Reaction Using (Hetero)Aryl Chlorides and Chemoselective Hydrogenation. *Adv. Synth. Catal.* **2017**, *359* (13), 2269–2279. https://doi.org/10.1002/adsc.201700156.

(17) Khatal, S. B.; Padmor, M. S.; Mariet, M.; Pratihar, S. Room Temperature Transfer Hydrogenation of Aldehydes Using Methanol Catalyzed by the Iridium(III) Pyridylidene–Indole Complex. *Chem. Commun.* **2025**, *61* (44), 8063–8066. https://doi.org/10.1039/D5CC00398A.

(18) Margarita, C.; Di Francesco, D.; Tuñon, H.; Kumaniaev, I.; Rada, C. J.; Lundberg, H. Mild and Selective Etherification of Wheat Straw Lignin and Lignin Model Alcohols by Moisture-Tolerant Zirconium Catalysis. *Green Chem.* **2023**, *25* (6), 2401–2408. https://doi.org/10.1039/D2GC04650D.
